# Enhancing Osseointegration of TC4 Alloy by Surficial Activation Through Biomineralization Method

**DOI:** 10.3389/fbioe.2021.639835

**Published:** 2021-02-23

**Authors:** Liang Zhou, Meng Pan, Zhenghua Zhang, Zijie Diao, Xiaochun Peng

**Affiliations:** ^1^Department of Materials and Engineering, School of Forestry and Landscape Architecture, Anhui Agriculture University, Hefei, China; ^2^Department of Thoracic Surgery, The First Affiliated Hospital of USTC, Division of Life Sciences and Medicine, University of Science and Technology of China, Hefei, China; ^3^Department of Orthopaedics, The Sixth Affiliated People’s Hospital, Shanghai Jiao Tong University, Shanghai, China

**Keywords:** silk fibroin, osseointegration, hydroxyapatite, surface modification, biomineralization

## Abstract

Titanium (Ti) alloys have been applied to biomedical implants for a long time. Although Ti alloys are biocompatible, efforts have been continuously made to improve their bone conductivity and osteogenesis for enhancing their performance. Silk fibroin (SF) is a natural biomaterial with excellent biomedical and mechanical properties, and hydroxyapatite (HAP) nanocomposites derived from SF are promising for producing “artificial bone” owing to their biomedical applicability and strong mechanical functions. Therefore, we built an SF coating on the surface of Ti–6Al–4V alloy, and then the incubated SF-coated Ti alloy were immersed in simulated body fluid to induce mineral deposition of HAP on the alloys. The results from Scanning Electron Microscopy (SEM), X-ray Diffraction (XRD) analysis, and Attenuated Total Reflection–Fourier Transform Infrared Spectroscopy (ATR–FTIR) confirmed the deposition of a mineral layer on the SF film surface. The proliferation, adhesion, and differentiation of MG-63 were tested, along with the BMP-2, COX-2, and OPG expression and protein content in the MG-63. Both Ti + SF and Ti + SF + HAP groups exhibited significantly better performance than a control Ti group with regard to the cell adhesion, cell proliferation, and protein expression. Furthermore, the hybrid layer comprising HAP and SF delivered more significant improvement of the osseointegration than the SF alone. It is hoped that the proposed methods can be used for constructing modified surfaces on Ti alloys, as they endowed the implants with good osteogenic potential.

## Introduction

Titanium (Ti) and Ti alloys are widely used in biomedical devices and components (particularly as hard tissue replacements), as well as in cardiac and cardiovascular applications, because of their desirable properties, such as their relatively low modulus, good fatigue strength, formability, machinability, and corrosion resistance (Liu et al., 2004). The chemistry and topography of implant surfaces are critical for the successful tissue integration of load-bearing dental and orthopedic implants. Additionally, the surface properties of Ti implants are key factors for rapid and stable bone tissue integration, i.e., osseointegration, which indicates the establishment of a durable, direct, and functional link between the bone and the implant surface. It is well known that grit blasting and acid etching are effective for improving the surfaces of dental implants (Salou et al., 2015). Physical modification via grit-blasting produces a rough surface at the microscale, which increases the degree of mechanical interlocking with the bone tissue (Longhitano et al., 2015). However, this type of rough surface has random and uncontrolled microstructures, while proteins and cells interact with the implant surface at the nanoscale. It has recently been shown that nanostructures on Ti implants produced by electropolishing significantly enhance the adhesion and osteoblastic differentiation of mesenchymal stem cells (Lausmaa, 1996; Harris et al., 2007; Lu et al., 2020). Nevertheless, the *in vivo* environment triggers the release of metal ions from the implant and then initiates malfunction of joint replacements and even toxic effects in the serum. Thus, several types of biocompatible coating, such as bioglass (Liu and Miao, 2004; Xue et al., 2017), collagen, and silk fibroin (SF) (Kim et al., 2005; Melke et al., 2016; Yu et al., 2017; Saha et al., 2019), have been introduced to suppress the release of metal ions from implants (Ku et al., 2002). However, the coating alone is inadequate for delivering a satisfactory roughness on the surface of metal alloys as other aforementioned physical treatments do. Thus, it is necessary to establish a biocompatible coating that can encapsulate a Ti implant and establish a topographical structure with depressions of <100 nm.

The biomineralization process is important for natural biological materials (Gu et al., 2015). It also provides an ecofriendly approach for forming artificial materials, which have a hierarchical structure comprising organic and inorganic elements. It was reported that the inert surface of the implant could be transformed into an activated surface by coating it with a mineral layer through biomineralization (Svensson et al., 2013). In particular, the topography of the layer has roughness at the nanoscale, which benefits both surface-to-blood interactions and osseointegration. SF, which is isolated from a natural fiber secreted by *Bombyx mori*, has been used as a biomedical material owing to its unique biocompatibility and minimal inflammatory reactions (Kumar and Singh, 2017). It has been approved as a biomaterial by the United States Food and Drug Administration (Kim, 2014). As an amphiphilic copolymer, regenerated SF can regulate the formation and growth of hydroxyapatite (HAP) and aragonite in an aqueous system via biomineralization (Das et al., 2018). Therefore, in present study, we prepared an HAP coating with roughness at the nanoscale on Ti alloys through a biomineralization process in which SF was used as an inducing template. The topographic features, chemical groups, and crystalline structure were compared between the original and coated Ti alloys. Then, the osseointegration performance of the alloys was evaluated according to observations of the adhesion and osteoblastic differentiation of mesenchymal stem cells and several important biological tests. The effect of the mineral coating on the application of Ti was discussed further when the characteristics of the implant surface were systematically related to the overall osseointegration performance in this paper. It is expected that the constructing of such coatings on Ti alloys is helpful for enhancing osseointegration of Ti based implants.

## Materials and Methods

### Materials

One of Ti alloys, Ti–6Al–4V, which was used as an implant in clinical surgery for >10 years, was selected as the origin material. Ti–6Al–4V disks with a diameter of 15 mm and a height of 5 mm were manufactured before further treatment. The samples were first wet ground with 120, 600, 1,200, and 2,400 grit silicon carbide abrasive paper at approximately 150 rpm (Ku et al., 2002). Then, a nitric acid passivation treatment based on the ASTM F86 protocol with 30% nitric acid was applied to the Ti alloy disks for 1 h to prepare a control sample labeled as Ti (Ku et al., 2002).

Silk fibroin solution was prepared according to previously reported methods, as follows (Qian et al., 2014): the silk cocoon was degummed in 0.5 wt.% NaCO_3_ aqueous solution for approximately 40 min to remove the sericin. The degummed silk was then dissolved in a 9.3 M LiBr aqueous solution. After being filtered, the fibroin solution was dialyzed against deionized water for 72 h at room temperature with a 12,000–14,000 molecular weight cutoff dialysis membrane to remove the salt. The dialyzed solution was then clarified via spinning in a centrifuge at 6,000 rpm for approximately 4 min. The supernatant SF solution was collected and diluted to 2 wt.% before being stored at 4°C. These Ti alloy disks were immersed in a 2% SF solution at room temperature for 2 h. Subsequently, they were dried in a desiccator with the atmospheric conditions preset as 20°C and 70% ± 5% relative humidity by laying them above a sodium chloride saturated solution. One week later, the disks were removed, vacuum-dried for 24 h at room temperature, and labeled as Ti + SF. HAP was deposited onto the Ti + SF disks using an alternate soaking process. The disks were immersed in a 200 mM CaCl_2_ solution in a Petri dish placed on a mechanical shaker with shaking at 150 rpm for 1 h at 37°C. The disk was then blotted onto filter paper to remove excess moisture and then immersed in a 120 mM Na_2_HPO_4_ solution for 1 h. The whole depositing process was repeated three times. And then, the disk was washed in distilled water, air-dried at room temperature for 24 h, and labeled as Ti + SF + HAP.

### Methods

#### Characterization of Samples

All three samples, i.e., Ti, Ti + SF, and Ti + SF + HAP, were observed via scanning electron microscopy (SEM, Tescan TS5136MM, Brno, Czechia) at an accelerating voltage of 20 kV after gold spouting to examine the surface morphology. The crystallization patterns of the three samples were recorded using a wide-angle X-ray diffraction (XRD) instrument (D8 Advance, Bruker AXS, Germany) with CuKα radiation (*λ* = 0.154 nm). The XRD data were collected from 2*θ* = 5° to 70° at a scanning rate of 2°/min. Attenuated total reflection–Fourier transform infrared (ATR–FTIR) measurements were performed using a Nicolet Nexus 6,700 spectrometer (Saxena et al., 2017). Each spectrum was recorded with 64 scans and a resolution of 4.0 cm^–1^. The spectra of Ti + SF and Ti + SF + HAP were analyzed in the range of 650–4,000 cm^–1^.

#### Cultivating Cell and Its Observation

MG-63 cells were purchased from Institute of Biochemistry and Cell Biology, China Academy of Science, and cultured in Dulbecco’s modified Eagle’s medium containing 10% fetal bovine serum (Sigma), 1% PSF (100×, 10,000 U/mL Penicillin, 10,000 mg/mL Streptomycin, and 25 mg/mL Fungizones) at 37°C in an incubator with 5% CO_2_. MG-63 cells were seeded to the three types of sterilized samples (Ti, Ti + SF, and Ti + SF + HAP) in 24-well plates and cultured under normal conditions. After 1, 4, and 7 d of cultivation, the cell adhesion and proliferation of the three Ti alloy samples were assessed via immunofluorescence observations using a confocal scanning laser microscope (CLSM, FV3000 OSR, Olympus, Japan). F-actin was stained with phalloidin (red). Nucleus staining was performed using DAPI (blue). The excitation of laser was set at 360 and 488 nm, respectively.

#### Cell Proliferation Activity Impact Assay

The MG-63 proliferation was measured via cell counting kit-8 (CCK-8) assays, as reported previously. MG-63 cells were seeded to the three samples (Ti, Ti + SF, and Ti + SF + HAP) in 24-well plates and cultured under normal conditions. After 8 h of culturing, the samples were moved to a new culture plate, and the culturing continued. Then, cells were added at different time points (1, 3, 5, and 7 d) with 10 μL of a CCK-8 solution (Dojindo, Japan) in each well, followed by culturing for another 4 h. Subsequently, 100 μL of the supernatants were transferred to a 96-well plate. All three samples with the MG-63 cells were rinsed with serum-free media, and the number of proliferative cells was quantified via a CCK-8 assay (Dojindo, Japan) according to the manufacturer’s instructions. The absorbance of each specimen was measured at 450 nm using a microplate reader (MultiSkan FC, Thermo, United States). All the experiments were performed in triplicate.

#### Alkaline Phosphatase (ALP) Activity Test

ALP is an early differentiation marker associated with calcification (Ku et al., 2002). Therefore, osteogenesis can be indicated by the expression of ALP. An ALP activity test was performed using a kit manufactured by Abcam, Alkaline Phosphatase Assay Kit (Colorimetric). MG-63 cells were cultivated on a 24-well cell culture plate, in which testing samples were preset, with a density of 5.3 × 10^3^ cell/cm^2^. We transferred the samples to new culture plates after 8 h of culturing. After 14 d of culturing, the cells and the upper supernatant of the cultivating suspension were collected for evaluating the ALP activity in the intercellular and extracellular environments, respectively. All experiments were performed in triplicate.

#### Intracellular BMP-2, OPG, and COX-2 Protein Content

After 5 d of cultivation on different testing samples, the cells were rinsed with 1 mL of cooled dPBS, and 1 mL of a cell lysis buffer (50 mM pH 8.0 Tris, 1 mg/mL leupeptin, 150 mM NaCl, 0.5% Non-idet P-40, 5 mM EDTA, 100 mM phenylmethylsulfonyl fluoride, 1 M dithiolthretol, and 1 mg/mL aprotinin) was added for protein extraction. A BCA assay was performed to determine the protein concentrations. Protein samples (10 μL/lane) were separated by 10% SDS-PAGE and transferred to PVDF membranes via the wet transfer method. Blots were blocked in TBST containing 5% non-fat milk at room temperature for 2 h and incubated with the primary antibodies (BMP-2, 1:300; OPG, 1:500; COX-2, 1:200; beta actin, 1:800) at 4°C overnight. The membranes were then rinsed with TBST and incubated with the secondary antibodies for 2 h. ECL chemiluminescence substrates and X-ray films were used to detect the bands, and the relative optical densities were analyzed using an image-processing software program: the relative contents of BMP-2 and OPG or the COX-2 optical density of the target band/optical density of beta-actin band. The results were normalized with respect to those for integrin.

#### Statistical Analysis

All experiments were performed in triplicate unless otherwise specified. A single-factor analysis of variance was performed to calculate the statistical differences, and *p* < 0.05 indicated statistical significance. The statistical analysis results were expressed as the mean ± standard deviation (SD).

## Results and Discussion

### Observation of Testing Samples

**FIGURE 1:**
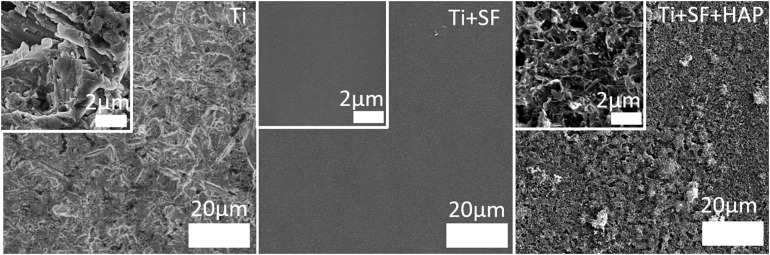
SEM images of the three Ti samples: the Ti alloy substrate (Ti), coated SF Ti alloy (Ti + SF), and HAP/SF hybrid coated Ti alloy (Ti + SF + HAP). The inset images are locally magnified 10×.

To examine and compare the surfaces of the Ti alloy substrate (Ti), Ti alloy coated with SF (Ti + SF), and coated Ti alloy after biomineralization (Ti + SF + HAP), SEM was performed. The images obtained are shown in Figure 1.

Clearly, the surface of the Ti alloy substrate was ravined, with roughness at the microscale. After the coating with SF, the surface became flat and uniform. The inset images indicated significant differences between the two surfaces. This indicates that the coating process covered the original Ti alloy substrate (Saha et al., 2019). Nonetheless, the rough surface returned after the biomineralization process. However, this time, the roughness was finer. Nanosized pores, needles, and plates, which are attributed to the HAP deposited during biomineralization, are observed in the inset image of Ti + SF + HAP. The nanoscale structures on the surfaces of the implanted materials were proven to be beneficial for cell adhesion, thereby promoting osseointegration (Svensson et al., 2013). Therefore, it is hoped that the cell adhesion will be improved after *in situ* biomineralization on the coating of SF above the Ti alloy substrate.

### Characterization of Testing Samples

**FIGURE 2:**
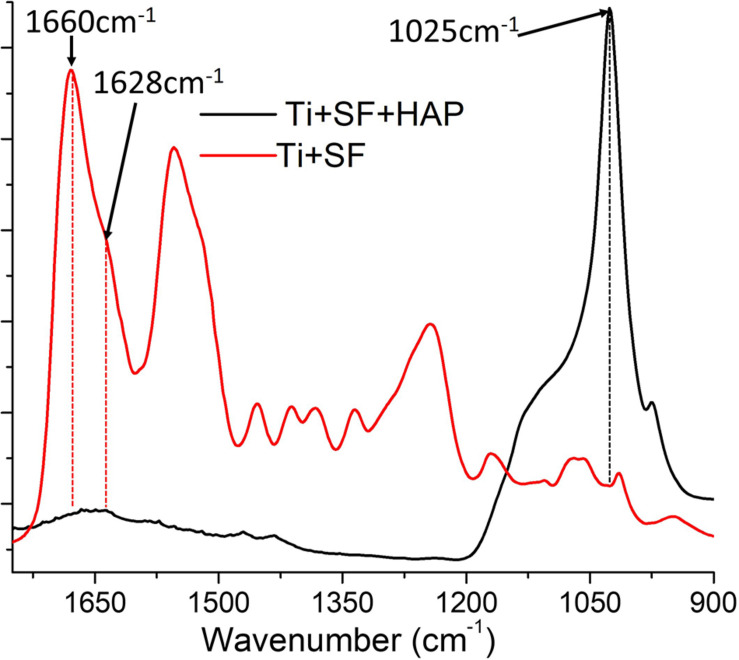
ATR–FTIR spectra of Ti + SF and Ti + SF + HAP samples.

To clarify the conformation of SF when it was coated onto the Ti alloy, FTIR spectroscopy was performed on the Ti + SF and Ti + SF + HAP samples. The results are shown in Figure 2.

The FTIR spectrum of Ti + SF resembled that of regenerated SF materials after enduring the treatment of methanol. The assignment of adsorption peaks in the amide I band of SF is generally agreed upon: the broad peak centered at 1,655–1,660 cm^–1^ is assigned to the random coil, helical conformation, or both; the peak at approximately 1,620–1,630 cm^–1^ is assigned to the β-sheet conformation; and the small peak near 1,700 cm^–1^ is assigned to the β-turn conformation of the hairpin-folded antiparallel β-sheet structure. The peak related to the conformation of the random coil was larger than the others. It is reported that slow drying at room temperature tends to induce the aggregation of molecular chains of SF aggregated into the random coil. Furthermore, a shoulder peak at approximately 1,628 cm^–1^ was observed. This indicates that the β-sheet conformation of SF was formed during the dehydration. Thus, SFs with various conformations were successfully immobilized on the Ti surface. The FTIR spectrum of Ti + SF + HAP exhibited a strong peak near 1,025 cm^–1^ due to the vibration of PO_4_^3–^. The typical SF absorption of FTIR was blocked by deposited HAP, and the peak became smaller or even disappeared. This indicates that the HAP was deposited on the SF coating and covered the surface of SF + Ti after biomineralization.

**FIGURE 3:**
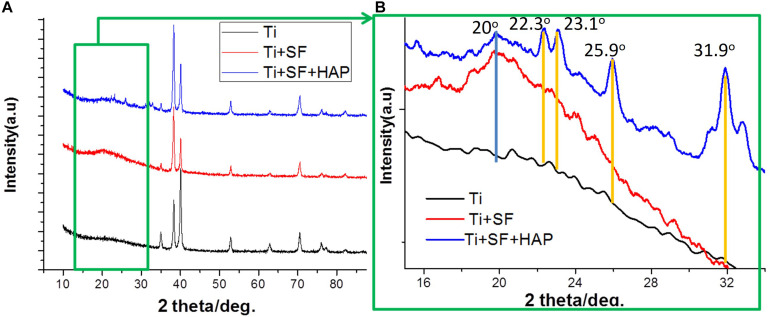
XRD patterns of Ti + SF and Ti + SF + HAP samples. Panel **(B)** is a local magnification of panel **(A)** (green box).

X-ray diffraction spectroscopy was performed to examine the crystalline structure of the substances on the substrate. Figure 3 presents the XRD patterns of the Ti, Ti + SF, and Ti + SF + HAP samples. As shown in Figure 3A, the 2*θ* peaks of the Ti substrate resembled the typical characteristic absorption peaks of the Ti alloy (Ti6Al4V). Because the X-ray peak corresponding to SF was significantly smaller than that of the metal alloys, the SF + Ti exhibited the same XRD patterns from 2*θ* = 33° to 90° after the SF coating. However, the local enlargement from 2*θ* = 15° to 35° in the pattern indicated the difference between the two samples. A new peak emerged at approximately 2*θ* = 20° for Ti + SF, which is generally assigned to the silk II crystalline structure. This indicates that the molecular chains of the SFs formed a crystalline structure on the surface of the Ti alloy after the dehydration. It is beneficial to stabilize the coating during biomineralization. Furthermore, after the biomineralization, several additional 2*θ* peaks emerged (at 22.3°, 23.1°, 25.9°, 31.0°, 31.9°, and 32.8°), which were consistent with the diffraction peak positions of HAP in the standard cards. The results suggest that the Ti alloy was successfully covered by the SF through the coating process. Then, the HAP/SF hybrid layer was successfully constructed via the biomineralization method.

### Observing Cultivation of Cells

**FIGURE 4:**
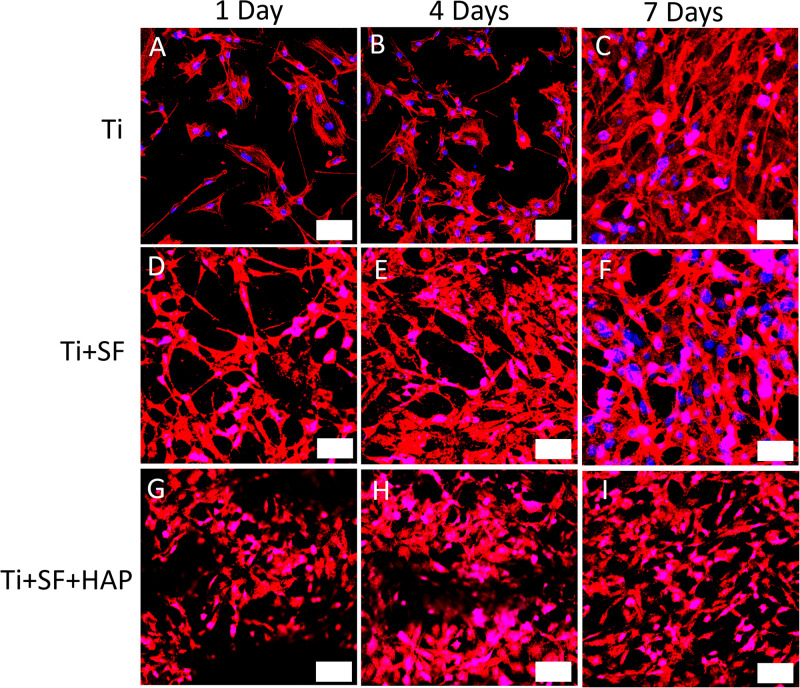
CLSM observations for immunostaining MG-63 cells on three different testing materials: Ti **(A–C)**, Ti + SF **(D–F)**, and Ti + SF + HAP **(G–I)** after 1 d **(A,D,G)**, 4 d **(B,E,H)**, and 7 d **(C,F,I)** of cultivation. The bar indicates 50 μm. Blue color shows the nucleus of MG-63 cells which was stained by DAPI and the red color demonstrates the F-actin which was stained with phalloidin.

Through a fluorescence staining analysis of CLSM, the adhesion and proliferation of MG-63 cells on the Ti, Ti + SF, Ti + SF + HAP samples after 1, 4, and 7 d of cultivation were examined, as shown in Figure 4. For the Ti group, a progressive increase in the cell proliferation was observed during the 7-d cultivation. The same trend was also detected in the remaining two groups. The 7-d observation results indicated that the cell adhesion and proliferation ability for the Ti + SF group were slightly improved compared with those for the Ti group, and the amount of cells for the Ti + SF + HAP group far exceeded those for the other two groups. Previous findings indicated that SF have the ability to promote cell adhesion and proliferation (Minoura et al., 1990). A better cell adhesion and proliferation was also found in the SF + Ti group than in Ti group according to our observation of CLSM. It is reported that the introduction of HAP is beneficial for promoting the growth of cells on the implant during osseointegration and osteogenesis (Zhu et al., 2012). This is evidenced by the CLSM observations for the Ti + SF + HAP sample compared with the Ti and Ti + SF samples in this work. Thus, both the SF coating and the subsequent biomineralization positively affected the cell adhesion and proliferation. Nevertheless, the biomineralization show a better effect than the SF coating alone. The results indicate that a large specific surface area of the implant is important for the adsorption of living cells. Hence, this difference between Ti + SF and Ti + SF + HAP was attributed to the nanoscale roughness of the HAP deposited layer after the biomineralization, as mentioned in section “Observation of Testing Samples.”

### Cell Proliferation, Differentiation, and Adhesion

**FIGURE 5:**
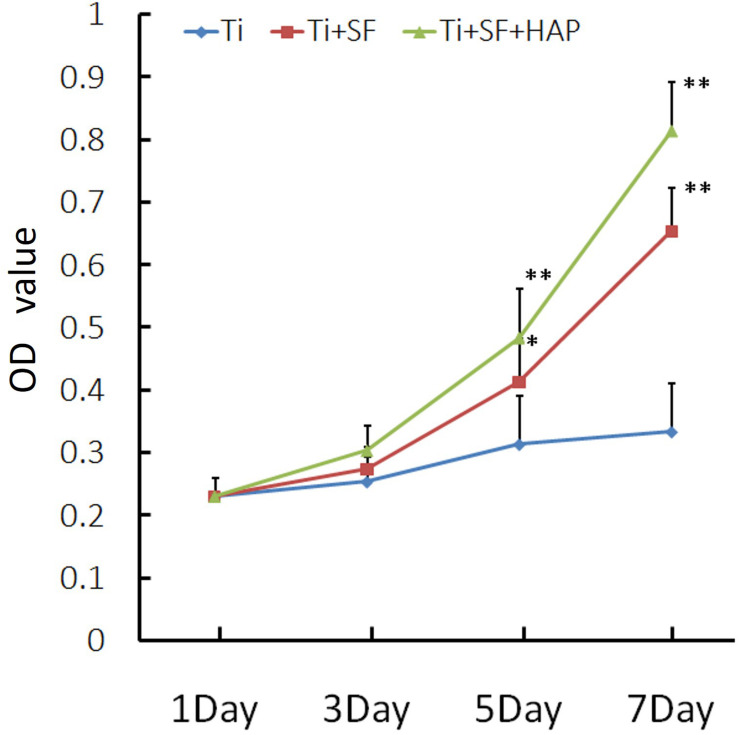
MG-63 proliferation was measured via a CCK-8 assay (^∗^indicates *p* < 0.05 and ^∗∗^indicates *p* < 0.01).

The cell proliferative activity of MG-63 cells on the three samples was evaluated via a CCK-8 assay. The results of the cell proliferative activity assay are shown in Figure 5. The MG-63 cells adhered to the three samples after inoculation. However, the cellular activity of the MG-63 cells was significantly higher for Ti + SF and Ti + SF + HAP than for Ti after 5 and 7 d of cultivation (*p* < 0.05). There was no significant difference between Ti + SF and Ti + SF + HAP at any of the detection time points. Thus, the cell adhesion and growth capacity of Ti + SF and Ti + SF + HAP were significantly better than those of Ti. The large roughness of the surface formed by biomineralization contributed to the microstructures and nanostructures on the surface, which improves protein adsorption (Maleki et al., 2019). This explains why the cell adhesion and growth capacity were significantly improved after the coating of Ti + SF + HAP.

**FIGURE 6:**
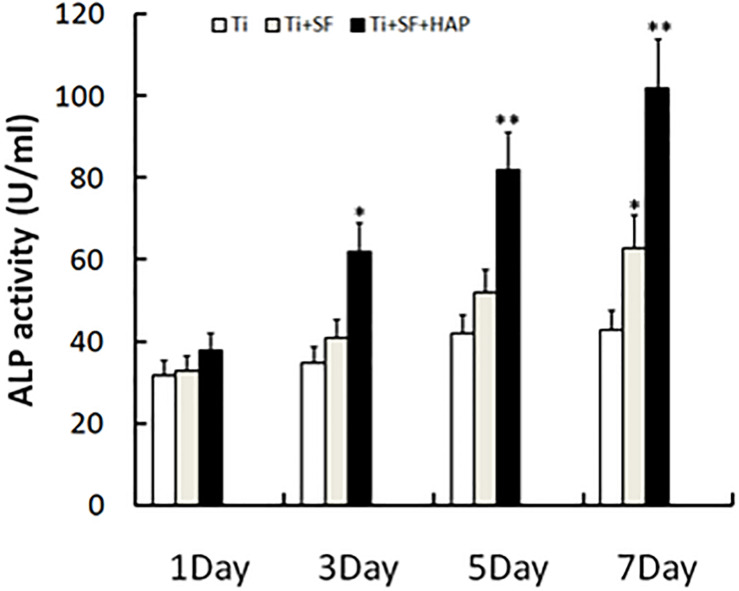
Cell differentiation was monitored by measuring the ALP activity (^∗^indicates *p* < 0.05 and ^∗∗^indicates *p* < 0.01).

ALP is the most widely recognized marker of osteoblast differentiation in the early stage and is essential for the maturation and calcification of osteoblasts. When osteoblasts actively deposit bone matrix, they secrete large amounts of ALP. Therefore, the expression of ALP can be used to prove the osteogenic effect (Cai et al., 2002). The ALP activity of MG-63 cells grown on the samples was measured 1, 3, 5, and 7 d after inoculation, and the results are presented in Figure 6. Ti + SF exhibited a significantly higher ALP activity than Ti after 7 d (*p* < 0.05). This indicates that the coating of SF onto the Ti alloy substrate improved the osteoblast differentiation ability. This finding is consistent with previous reports stating that SF can support the attachment and proliferation of osteoblasts, as well as the attachment and differentiation of osteoclasts (Qian et al., 2014). Furthermore, Ti + SF + HAP exhibited a significantly higher ALP activity than Ti at 3, 5, and 7 d (*p* < 0.05 or *p* < 0.01). It suggests HAP/SF hybrid layer can promote the osteoblast differentiation on the 3rd day after inoculation. The results indicate that the SF + HAP coating prepared via the biomineralization process enhanced the osteoblast differentiation.

**FIGURE 7:**
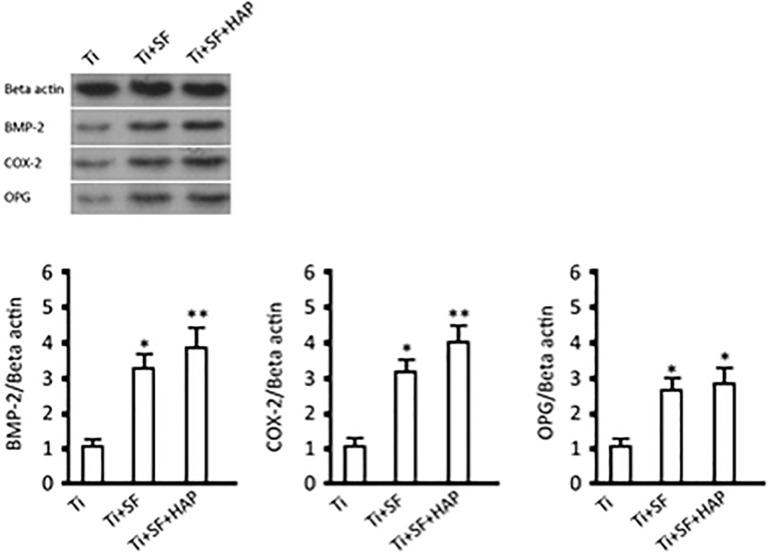
BMP-2, COX-2, and OPG expressions and protein contents (^∗^indicates *p* < 0.05 and ^∗∗^indicates *p* < 0.01).

The morphogenesis and remodeling of bone depends on the integrated activity of osteoblasts, which form bone, and osteoclasts, which resorb bone (Olszta et al., 2007). BMP-2 is one of the most important bone formation stimuli that can induce osteoblastic differentiation (Wang et al., 2014). Additionally, BMP-2 is the most important regulator of OPG protein expression in osteoblasts (Xia et al., 2015). OPG is considered to be a key cytokine that mediates the formation and activity of osteoclasts and specifically inhibits the development of osteoclasts. Cyclooxygenase-2 (COX-2) is involved in the inhibition of apoptosis and the enhancement of cell growth (Katagiri et al., 2006). Therefore, expressions of these proteins were selected to illustrate the activity of osseointegration of MG-63 cells on three samples, and the results are shown in Figure 7. The expressions of all the testing proteins exhibited significant differences between the Ti samples and the Ti + SF samples (*p* < 0.05). Meanwhile, a larger difference was observed between the Ti samples and Ti + SF + HAP samples, as the statistical difference level was 0.01 for the proteins of BMP-2 and OPG. The foregoing results indicate the capacity of osteoinduction to promote osteogenesis in the Ti + SF + HAP group. Mineral-based biomaterials with osteoconductive and potential osteoinductive properties have been proven to promote osteogenesis (Zhao et al., 2009). Considering that the hybrid coating layer has similar chemical properties and structures to the natural bone mineral, it can be considered as a biomimetic mineral (Qian et al., 2014). It clearly be proved by our results which suggested the hybrid coating promotes osteogenesis of Ti alloys substrate.

## Conclusion

Osseointegration is mainly affected by the surface properties of Ti implants, such as the surface composition and wettability. Therefore, we successfully introduced SF and HAP coatings on Ti alloy substrates via a stepwise deposition process. The results indicated that the SF coating wrapped the substrate and the nanoscale HAP coating was subsequently formed via *in vitro* biomineralization. Several biological tests were performed to evaluate the activity of MG-63 cells on three different implants: Ti, Ti + SF, and Ti + SF + HAP. A comparison of the activity among these implants suggested that both the coatings promoted the adhesion, proliferation, and differentiation of MG-63 cells. Furthermore, the hybrid layer comprising HAP and SF delivered a more significant improvement of the osseointegration than the SF alone. The proposed method for preparing a hybrid coating on the Ti alloys substrate can be used to improve the osseointegration of Ti alloy implants in the future.

## Data Availability Statement

The original contributions presented in the study are included in the article/supplementary material, further inquiries can be directed to the corresponding author.

## Author Contributions

LZ done the experiment design and manuscript preparation with MP. ZZ taken the job of cell cultivation and proliferation activity impact assay. ZD was responsible for characterizing the samples. XP supervised the work and together with LZ and MP wrote the publication. All authors contributed to the article and approved the submitted version.

## Conflict of Interest

The authors declare that the research was conducted in the absence of any commercial or financial relationships that could be construed as a potential conflict of interest.
